# Supplementary material for: An evidence-based guidance framework for neural network system diagrams
Source: PLoS One. 2025 Mar 18;20(3):e0318800. doi: 10.1371/journal.pone.0318800 (PMC12366753; doi:10.1371/journal.pone.0318800)
Supplement: Attachment — Submitted filename: PLOS_ONE_Rebuttal_Reviewers.pdf [file pone.0318800.s002.pdf]

4 November 2024

Dear Editor and Reviewers,

Thanks to the two reviewers for their constructive feedback. Responding to each point in turn.

Reviewer 1:

1. Could the authors provide more details on the experimental design, particularly the specific steps of data collection and analysis? *A method overview has been added (p13). Additionally, information has been added about tools used throughout the reporting, e.g. p14: "The interview transcripts were imported and themes labelled using NVivo 12 qualitative data analysis software" ". Throughout the method for the framework evaluation, the tooling used has now been specified (email, Qualtrics, NVivo, R, Excel).*

2. Given the diversity of diagram designs, how do the authors ensure the general applicability of the proposed framework? *It is hoped that the corpus analysis performs this check at a broader scale. Along these lines, it is not claimed that this is a fully generalisable framework. To make the limits of our claim clearer, we have added a further limitation on p19 (This is a small scale, in depth study, and results may not be generalisable to neural network subdomains which are not specialisms of participants.) and on p46 (This corpus analysis suggests that the framework may be generalisable to cases beyond the interview study and experimental framework evaluation.).*

3. Do the authors plan to further expand this study, for example, by incorporating more baseline comparisons or broader field applications? *In spring 2024, an interdisciplinary workshop was held at University of Manchester to progress the topic of technical diagramming, particularly in AI. A dozen interested parties took part, and the output of this is still emerging. In the short term, aidiagrams.com is promoting this topic. Further research is expected to be published within the Diagrams community, as are invited talks (e.g. at IDIAP in spring 2025).*

Reviewer 2:

1. Could the authors clarify why the study predominantly focuses on CV and NLP topics, given that only one participant out of 12 in Table 1 is from an AI or science background? Since the paper addresses neural network systems, further exploration and discussion of other NN-related domains beyond CV and NLP would be beneficial. *CV and NLP were chosen to constrain the scope for both the interviewee inclusion criteria and for the corpus analysis. Having said that, the labels in Table 1 may be misleading; the applicants all had specialisms (e.g. Oncology, or cultural institution management) but chose to identify with*

*the CV or NLP community in the context of this study (where the inclusion criteria was to have submitted to one of those venues). This is addressed as part of the experimental design clarifications for R1.*

2. In Chapter 4, the authors rely heavily on statements from 12 participants. I question whether this method is entirely appropriate, as the responses may contain excessive subjective bias. A more in-depth discussion of the validity of this experimental approach, along with supporting citations, would strengthen the paper. *A new limitations section has been added on p15, including comments on selection bias. However, the responses are indeed subjective. The point of an interview is to understand an individual's perspective, so their data cannot be biased per se. The issue with bias in this methodology would be leading questioning or poor schedule design, which we do not believe you are suggesting. If we had interviewed different people we may have got different results, but the results of this interview study are valid, as evidenced by the evaluation.*

3. The first-line indentation is inconsistent throughout the paper; some sections have indentation, while others do not. *Apologies if this is distracting. Indents have been added, and bold paragraph titles have been replaced by bold text, adjusting the spacing.*

4. There are formatting issues with the references, including but not limited to L164, L217, and L228. *Page numbers have been removed from references, resolving this confusion.*

We hope reviewers are satisfied that the changes above make this paper fit for publication. Thank you once again for your reviews and kind attention to this cover letter and to the paper.

Best wishes

Guy Marshall,
André Freitas,
and Caroline Jay
