# Supplementary material for: An evidence-based guidance framework for neural network system diagrams
Source: PLoS One. 2025 Mar 18;20(3):e0318800. doi: 10.1371/journal.pone.0318800 (PMC12366753; doi:10.1371/journal.pone.0318800)
Supplement: Attachment — Submitted filename: review comments.docx [file pone.0318800.s001.docx]

This paper studies the diverse uses and understandings of scholarly neural network system diagrams, proposing an improvement framework based on existing design, information visualization, and user experience principles. Through a combination of interviews, card sorting, and qualitative feedback, the research reveals the diversity and individual preferences in creating and interpreting these diagrams. Additionally, the paper evaluates the effectiveness of the framework through a mixed-methods experimental study and a "corpus study" of published diagrams, aimed at enhancing the communicative efficiency of scholarly neural network diagrams.

**Paper Strengths**

1. Innovative research methodology: The article employs a mixed-methods research design that combines qualitative and quantitative approaches to provide a comprehensive analysis of the use of neural network system diagrams in academia.
2. Integration of theory and practice: By using ecologically-derived examples combined with theoretical analysis, the study systematically improves existing neural network system diagrams, enhancing the efficiency of information transmission in diagrams.
3. Thorough literature review: The article provides a solid theoretical foundation for its research design and results analysis by thoroughly reviewing relevant studies in the field.

**Paper Weaknesses**

1. Insufficient experimental details: The paper lacks detailed descriptions of the experimental setup and parameter adjustments, which may affect the reproducibility of the results.
2. Need for clearer exposition: Some sections of the paper, especially in methodology and results interpretation, are not clearly articulated, which may hinder readers' understanding.

**Questions to Authors and Suggestions for Rebuttal**

1. Could the authors provide more details on the experimental design, particularly the specific steps of data collection and analysis?
2. Given the diversity of diagram designs, how do the authors ensure the general applicability of the proposed framework?
3. Do the authors plan to further expand this study, for example, by incorporating more baseline comparisons or broader field applications?

**Overall,t**his paper proposes a valuable research framework, offering improvements for the design and interpretation of neural network system diagrams in academia. Despite some issues with experimental details and clarity of exposition, the methodological approach and comprehensive review of existing literature enhance its academic value. Based on the above, it is recommended that the paper be accepted after revisions.
